# Polyanhydride micelles with diverse morphologies for shape-regulated cellular internalization and blood circulation

**DOI:** 10.1093/rb/rbw047

**Published:** 2017-02-04

**Authors:** Guang Yang, Jie Wang, Dan Li, Shaobing Zhou

**Affiliations:** Key Laboratory of Advanced Technologies of Material, Minister of Education, School of Materials Science and Engineering, Southwest Jiaotong University, Chengdu 610031, China

## Abstract

Biodegradable amphiphilic poly (ethylene glycol) (PEG) based ether-anhydride terpolymer, consisting of PEG, 1, 3-bis (p-carboxyphenoxy) propane (CPP) and sebacic acid (SA), namely PEG-CPP-SA terpolymer, was employed to self-assemble into micelles by adding water into a solution of the terpolymer in tetrahydrofuran (THF). The shape of polyanhydride micelles can be regulated by simply adjusting the water addition rate, where spherical, rod-like and comb-like micelles can obtained under water addition rate of 20, 3 and 1 ml/h, respectively. The effect of micellar morphologies on the cellular internalization and intracellular distribution were characterized qualitatively with cervical cancer cells (HeLa cells) and hepatoma cells (HepG2 cells) by fluorescence microscopy, confocal laser scanning microscopy (CLSM), flow cytometry (FCM) and transmission electron microscopy (TEM). The results reveal that the cellular uptake of micelles are micelle-shape-dependent (rod-like micelles may possess the highest cellular internalization rate) and cell-type-specific. Each endocytic pathway can make a contribution to this process in different degree. Moreover, blood circulation experiments of these micelles were carried out, demonstrating that comb-like micelles have a relatively longer blood circulating feature, which may due to its irregular shape help to increase the sensitivity to fluid forces and allows them to tumble and align with the blood flow.

## Introduction

Scientists have long been working on finding effective ways to improve systemic chemotherapy for cancers to be more efficient and safety. With the development of nanotechnology, nanocarriers have been a recent research trend in systemic chemotherapy, which possess the ability of delivering the cargos preferentially into cancer tissues or targeted location, enhancing the therapeutic efficacy or transfection efficacy and reducing side effects [1, 2]. However, though with the help of nanocarriers, only less than 10% drug of total injected dose can reach and accumulate in the tumor tissue [3]. Efforts have been made to redesign these nanocarrirers to remedy this situation, such as optimizing the nanoparticle size [4, 5], modifying the surface chemistry of nanoparticle [6], functionalizing the nanocarriers [7–10] and regulating the shape of nanoparticles [6, 11, 12]. Among those strategies, regulating the shapes or geometries of the nanocarriers was emerging as a promising strategy to understand the internalization process and enhance the cellular internalization, prolong blood circulation and improve the biodistribution of nanoparticles [13–15]. For example, Rachit Agarwal *et al.* [16] have used nanorods and nanodiscs to evaluate the effects of particle geometry on the cellular uptake, and found that the uptake was particle-shape-dependent and governed by a combination of cell–particle adhesion, strain energy for membrane wrapping around the particle, and local particle concentration at the cell membrane. Huang *et al.* [12] have studied the cellular uptake by employing mesoporous silica nanoparticles with different aspect ratios (ARs, 1, 2, 4) and demonstrated that particles with larger ARs had faster internalization rates and larger internalization amounts. Yang *et al.* [17] have revealed that long nanorods would align to the cell membrane in a near-parallel manner followed by rotating by ∼90° to enter the cell via a caveolae-mediated pathway. In addition, Both Decuzzi *et al.* [18] and Li *et al.* [19] have reported that longer blood circulation duration and specific biodistribution were observed for disks than spheres. However, some researchers have also shown that spherical Au NPs possessed much faster cellular internalization compared to Au nanorods. These results suggest that a carefully scrutinize of the respective contribution of nanocarrier shapes, chemical compositions, and cell types are need to be considered when choosing a drug carriers.

Among those nanocarriers, polymeric micelles, self-assembled from amphiphilic block copolymers, were extensively explored and considered as a promising tool for drug delivery [20–22]. In our previous work, the polyanhydride micelles with good stability have successfully been fabricated from the polyether-based polyanhydride terpolymer PEG-CPP-SA [23, 24]. Moreover, the terpolymer can self-assemble into micelles with diverse morphologies (spherical, rod-like and filamentous shapes) in water by altering the ratio of PEG and SA in polymer backbone [23]. While, here, we have employed a more simple self-assembly method [25] to fabricate the multiple architectures. In this study, by fixing the weight feed ratio of PEG, SA and CPP as 30:50:20 in this polymer, we fabricated three types of micelles with different self-assembled architectures (spherical, rod-like and comb-like) via simply tuning the water addition rate during their self-assembly process. These three types of self-assembled architectures provide prototype models for shape-regulated cellular internalization and blood circulation.

## Materials and methods

### Materials

Poly(ethylene glycol) (PEG, *M_n_* = 2000), 1, 3'-dibromopropane, *p*-hydroxybenzoic acid and sebacic acid (SA) were bought from Chengdu KeLong Chemical Reagent Co. Nile red (Acros) was obtained from Shanghai Titan technology Co. LTD. 4',6-diamidino-2-phenylindole (DAPI) and amiloride hydrochloride (Amil) were purchased from Roche and Sigma, respectively. Chlorpromazine hydrochloride (CPZ) and genistein (Geni) were obtained from TCI and Adamas-beta, respectively. All other chemicals were of analytical grade.

### Cell lines and culture conditions

Human cervical cancer cell line (HeLa), Human liver hepatocellular carcinoma cell line (HepG2) and osteoblasts cell line (OB) were purchased from Sichuan University (China). HeLa and HepG2 cells were cultured in RPMI 1640 supplemented with 10% newborn calf serum (NCS, Gibco, USA).OB cells were cultured in α-MEM medium. All cells were carefully cultured in a humidified incubator at 37°C in an atmosphere of 95% air and 5% carbon dioxide.

### Synthesis of PEG-based ether-anhydride terpolymers

Briefly, the final PEG-based ether-anhydride terpolymers PEG-CPP-SA was synthesized via classical melt-condensation polymerization as stated in the previous reports [23, 24]. Firstly, prepolymer, 1, 3-Bis-(carboxyphenoxy) propane (CPP), was prepared according to our previous works [23]. Then, preweighed PEG, SA and CPP were mixed in a weight feed ratio of 30:50:20 in a three-neck round-bottom flask. The flask was then immersed in an oil bath under 180°C. After the prepolymers were completely melted, high vacuum was applied. The reaction was stopped after 2 h and the mixtures were allowed to cool completely and dissolved in dichloromethane. At last, the copolymers (PEG-CPP-SA) were precipitated using anhydrous ether and dried under vacuum with yields about 70%. All products were stored at −20 °C for further use.

### Formation of different architectures of micelles

Different architectures of micelles fabricated from amphiphilic PEG-based ether-anhydride terpolymer (PEG-CPP-SA) were prepared by adding water into a solution of PEG-CPP-SA in tetrahydrofuran (THF) with varied water addition rate. Typically, to obtain spherical, rod-like and comb-like micelles, PEG-CPP-SA terpolymers or terpolymers/Nile red mixture were first dissolved in THF (2 ml), then deionized water (4 ml) was added dropwise at a rate of 20.0 ml/h, 3.0 ml/h, 1.0 ml/h via a syringe pump at 20°C, respectively. After the THF was evaporated completely, sizes and polydispersities of these architectures were measured by Dynamic light scattering (DLS) measurements (Malvern Zetasizer Nano-ZS90 apparatus). The solution of the Nile red-labeled architectures were centrifuged at 1200 rpm for 4 min to remove the unloaded hydrophobic free Nile red and the upper layer of the solutions were gathered for further analysis.

### Characterization

Fourier transform infrared spectrometer (FT-IR) spectroscopy and Nuclear magnetic resonance (^1^H NMR) spectroscopy were applied to confirm the composition and structure of terpolymer (PEG-CPP-SA). Briefly, FT-IR spectrum was obtained on a Nicolet 5700 spectrometer by KBr sample holder method. ^1^H NMR spectrum was recorded on a Bruker AM 300 apparatus with CDCl_3_ as solvents and TMS as an internal reference.

DLS measurements were performed on a Malvern Zetasizer Nano-ZS90 detector at a copolymer concentration of 1.0 mg/mL. The labeled Nile red content was quantified by fluorospectro photometer (F-7000, Hitachi, Japan). Atomic force microscopy (AFM) (CSPM5000, China) with tapping-mode and transmission electron microscopy (TEM) (H-600, Hitachi, Japan) with operation at 75 kV were employed to observe the morphologies of the three architectures of blank micelles. For AFM and TEM observation, the concentrations of micelle solution were 0.5 mg/mL and 1.0 mg/mL, respectively. The Nile red-labeled micelles were observed by confocal laser scanning microscope (Leica TCS SP5).

### *In vitro* cytotoxicity evaluation

To evaluate the cytotoxicity of blank micelles, HeLa, HepG2 and OB cells were employed. The cells were placed in a 48-well plate for 24 h (1 × 10^4^ cells/well). After 24 h’s culture, freshly prepared three types of micelles solutions at different concentrations (10–200 μg/ml) were added to the culture medium. After incubation at 37°C for another 24 h, the medium was carefully removed and cells were rished with PBS. Then 300 μl Alamar blue solution (10% Alamar blue, 10% NCS and 80% media 199 (Gibco), v/v) was added into the each well and incubation for 4 h. After that, a sample of 200 ml of Alamar blue solution was transferred into a 96-well plate and the absorbance wavelength of each well on a microplate reader (ELX800, Biotek, USA) was set at 570 nm (excitation)/600 nm (emission) for the Alamar blue assay. To directly visualize, cells were stained with 1 μM calcein AM (Sigma, USA) and observed by fluorescence microscopy (FV1000, Olympus, Japan).

### *In vitro* cellular uptake

Fluorescence microscopy and confocal laser scanning microscopy (CLSM) were both employed for the cellular uptake observation. HeLa or HepG2 cells were seeded at 5 × 10^4^ cells/well into a 6-well plate and allowed to adhere overnight. Then freshly prepared three types of Nile red-labeled micellar solutions were added at a final Nile red-equivalent concentration of 10 μg/mL, respectively. After incubating for 30 min, 1 h, 3 h, or 6 h, the media were removed, and the cells were washed twice with PBS, fixed with 2.5% glutaraldehyde for 40 min and stained with DAPI for 5 min, and then visualized and photographed under a fluorescent microscope (IX51, Olympus, Japan).

The cellular ultrathin sections were also obtained and analyzed by TEM using the uranyl acetate/lead citrate double staining staining technique. Briefly, after cultured with three types of micellar solutions at a final concentration of 0.5 mg/ml, the HeLa cells were fixed with 3.0% glutaraldehyde and collected by high-speed centrifugation (15 000 rpm, 15 min) to tightly turn into blocks. Then, the blocks were dehydrated, embedded epoxy resin and sectioned into slices about 50 nm. Subsequently, after stained by uranyl acetate/lead citrate, the sections were mounted for observation by TEM.

### Inhibition study for the cellular uptake mechanism

HeLa and HepG2 cells were chosen to the study of endocytic pathways involved in the internalization of the three different micelles. Three endocytosis inhibitors, namely, chlorpromazine hydrochloride (CPZ, clathrin-mediated endocytosis inhibitor), genistein (Geni, caveolin-mediated endocytosis inhibitor), and amiloride hydrochloride (Amil, macropinocytosis inhibitor) were used to determine the cellular uptake mechanism. The inhibition studies of endocytosis were performed as follows: HeLa or HepG2 cells were seeded at 5 × 10^4^ cells/well into a six-well plate and allowed to adhere overnight, Then, the cells were pretreated with CPZ (10 μg/ml), Geni (50 μg/ml), Amil (50 μM) for 1 h, respectively. Subsequently, freshly prepared three Nile red-labeled architectures solutions were added at a final Nile red-equivalent concentration of 10 μg/ml to incubate for further 3 h. To inhibit the energy-dependent endocytosis, HeLa or HepG2 cells were pre-incubating at 4°C for 1 h and then followed incubation with the three different micelles for another 3 h at 4ºC. After that, the medium was removed, and cells were washed with PBS twice, fixed with 2.5% glutaraldehyde for 40 min and stained with DAPI for 5 min, and then visualized and photographed by the fluorescent microscopy and CLSM. The fluorescence intensity of the CLSM images were used to calculate the relative cellular internalization rate by using Plus 6.0 software.

For flow cytometry analysis, the cells were washed twice with cold PBS after desired time, followed by trypsinization and centrifugation at 1200 rpm for 4 min to gather the cells, then 1 × 10^4^ cells were gated and analyzed for fluorescent intensity with the flow cytometer (Accuri C6, BD, USA).

### *In vivo* blood circulation experiments

Healthy Balb/c male mice (20 ± 2 g, around 6 weeks’ old) were purchased from the Experimental Animal Center of Sichuan University. All Animals were feed in the condition of 25°C and 55% relative humidity. Three types of micelles fabricated from PEG-CPP-SA (7 µg Nile red-equivalent dose per kg) were injected intravenously with 100 μl solution in saline, respectively. At different time interval, orbital vein bleeds (∼0.1 ml) were obtained and centrifuged at 3000 rpm for 15 min to isolate plasma, the plasma was frozen at −20°C until analysis. The final Nile red content in blood was quantified by fluorospectro photometer (F-7000, Hitachi, Japan) against the standard curve. The percent injected dose (% ID) values and blood circulation half-life (t_1/2β_) were calculated as shown in a previous work [26].

## Results and discussion

### Synthesis of PEG-based poly (ether-anhydride) terpolymers

The composition and structure of poly(ether-anhydride) terpolymers (PEG-CPP-SA) were confirmed by FT-IR and ^1^H NMR as shown in Supplementary Fig. S1. The characteristic absorption bands of anhydride carbonyl (1810 cm ^−1^, 1741 cm ^−1^), ether bond (1083 cm ^−1^) and methylene (2929 cm ^−1^) are shown in the FT-IR spectra. Furthermore, peaks in the ^1^H NMR spectra (Fig. S1b) were consistent with our previous reports [9, 23, 24], which suggesting the successful synthesis of the PEG-CPP-SA.

### Fabrication of multiple architectures from PEG-based ether-anhydride terpolymers

**Figure 1:**
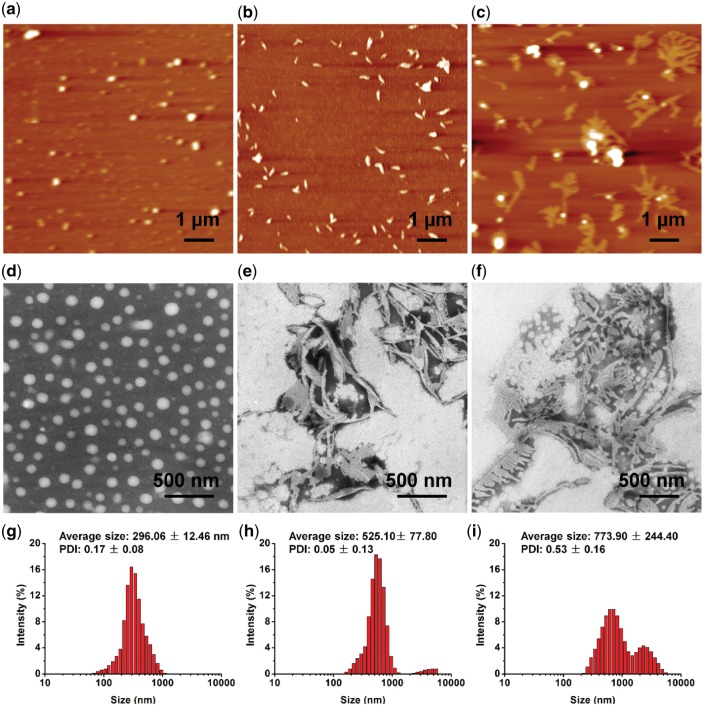
The morphologies of different shaped polyanhydride micelles characterized by AFM (**a**–**c**) and TEM (**d**–**f**) and the corresponding size distribution (**g**–**i**), including: a, d, g) the spherical micelles; b, e, h) the rod-like micelles and c, f, i) the comb-like micelles.

The micelles with diverse morphologies (spherical, rod-like and comb-like shapes shapes) were formed in water through a simple self-assembly method [25] from PEG-CPP-SA terpolymers with a fixed weight feed ratio of PEG, SA and CPP as 30:50:20. By changing the addition speed of water into the PEG-CPP-SA solution in THF, the spherical (20.0 ml/h) (Fig. 1a and d), rod-like (3.0 ml/h) (Fig. 1b and e) and comb-like (1.0 ml/h) (Fig. 1c and f) micelles were obtained. In this method, water was added into the organic phase, which is contrary to the usual way, adding the organic phase into the water. We can find that the spherical micelles were just like the normal micelles reported by many researchers, while the rod-like micelles seemed like a short rod with two sharp ends, and the comb-like micelles, a relative longer main micellar body with several shorter branches, looked like combs or tree branches. The DLS measurement was also applied to reveal the corresponding hydrodynamic radius distributions of spherical (Fig. 1 g, average diameter: ∼ 296.06 ± 12.46 nm), rod-like (Fig. 1 h, average diameter: ∼ 525.10 ± 77.8 nm) and comb-like (Fig. 1i, average diameter: ∼ 773.90 ± 244.4 nm) micelles. It seems that size of the micelles in the AFM and TEM images is smaller, which may due to the fact that the AFM and TEM were applied in a dry state of micelles, leading to shrinks of micelles. The confocal laser scanning microscopy (CLSM) was also used to observe the Nile red-labeled micelles in a wet state, as shown in Supplementary Fig. S2. The size of the micelles seem to be larger than in the AFM and TEM images, which may due to expansion of micelles in water and the refraction of lights, and the shapes of micelles look a little bit different. The spherical micelles (Supplementary Fig. S2a) were remain the same shape but larger, while the rod-like micelles (Supplementary Fig. S2b) were become more like short rods or ellipsoids, and moreover, the branches of the comb-like micelles (Supplementary Fig. S2c) were hard to distinguish owing to the resolution of CLSM.

**Scheme 1:**
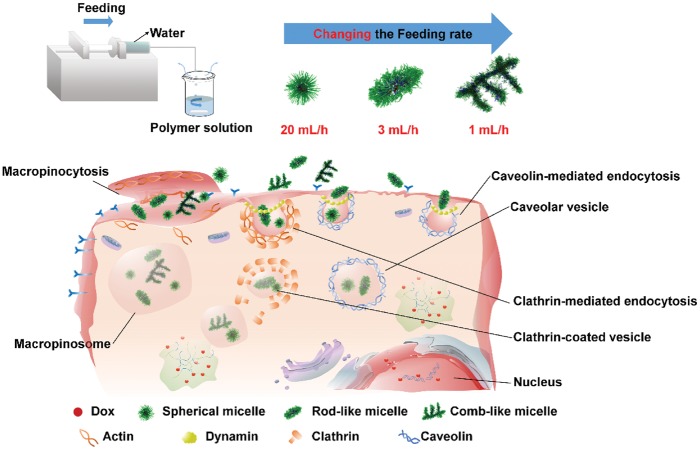
Schematic illustrations of polyanhydride micelles with diverse morphologies and their shape-dependent internalization.

Addition of water into the solution of PEG-CPP-SA terpolymers in organic solvents have triggered the self-assembly process. The morphologies of micelles can be affected by various factors, e.g. polymer concentration, cosolvent composition, temperature, and water content and addition rate [27]. Here, the variation is only the water addition rate, which plays a main role on self-assembling different architectures of block copolymer micelles. Under different water addition rates, the water content per unit of time is different at the initial stage in the mixed solution. A slower water addition rate should result in a lower water content per unit of time. Group of Eisenberg [27, 28] have reported that the kinetics of the morphological transitions are found to be very sensitive to the water content. They found that a morphological transition usually takes minutes to complete at 5.5% water content, but needs days at 8.5% water content, which suggests that the structures of the aggregates in the solvent mixture with a water content in the range used in the present study can very easily become kinetically locked when more water is added [28]. According to their report [28] at a relative low initial water content, the morphology of the aggregates is mainly controlled by thermodynamics of the micellization, which reflects a force balance between the repulsive interactions of the corona chains, the interfacial energy of the core/shell region, and the deformation of the hydrophobic polystyrene (PS) blocks in the core. In this condition, increasing the water content can promote the morphological transitions at some point when the degree of stretching of the PS blocks in the core has increased to some critical value of the extension. Hence, in our study, water addition rate of 1 ml/h or 3 ml/h supplied a relatively lower water content in initial, which is helpful to induce the micellar morphological transitions, and relative lower additional rate also give the micelles a break to complete the transition and then make the transformed morphologies get locked with continuous addition of water. While water addition rate of 20 ml/h may result in a too high water content at initial time to induce the morphologies transition.

### *In vitro* cytotoxicity evaluation

Supplementary Fig. S3 showed the viability and representative fluorescence images (live cell staining) of HeLa, HepG2 and OB cells incubated with the three types of blank micelles. After 24 h incubation, all cells remained a viability of more than 85%, even at a high concentration of 200 µg/mL of each blank micelle. Moreover, the representative live cell staining images showed that their growth is healthy, suggesting that the blank micelles (the copolymer) is non-cytotoxic.

### *In vitro* cellular uptake

**Figure 2:**
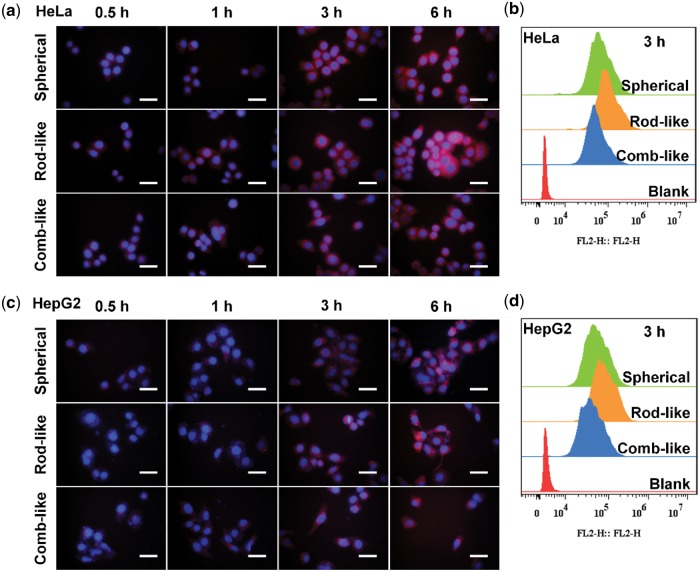
Cellular internalization. **(a)** fluorescence images of HeLa cells incubated with spherical, rod-like and comb-like micelles for 0.5, 1, 3, 6 h and **(b)** the corresponding flow cytometry histograms at 3 h. **(c)** fluorescence images of HepG2 cells incubated with spherical, rod-like and comb-like micelles for 0.5, 1, 3, 6 h and **(d)** the corresponding flow cytometry histograms at 3 h. Scale bar = 50 µm.

To investigate the effect of micellar morphologies on cellular uptake, Nile red-labeled spherical, rod-like and comb-like micelles were incubated with HeLa and HepG2 cells for 0.5, 1, 3 and 6 h. Then they were observed by fluorescence microscopy. As shown in the fluorescence images of Fig. 2a and c, we can see that the cellular uptake of the three types of micelles, in both HeLa and HepG2 cells, showed a time-dependent increase trend, during the observation time. Then the cellular internalization rates at 3 h were further confirmed by the flow cytometry (Fig. 2b and d). It seems that the rod-like micelles exhibit a higher internalization rate than other two morphological types of micelles, in both HeLa and HepG2 cells. While the comb-like micelles displayed the slowest internalization rate against both cells.

As previously reported, morphology [23, 25], size [29–31], surface functionalities [32, 33] and polymer composition [34] of nanoparticles will all affect their interactions with cell membranes and consequently cellular uptake. It has reported that rod-like or worm-like particles have a faster internalization rate than the sphere micelles, which may be owing to the larger contact area with cellular membrane than spherical nanoparticles [12, 16, 35]. Hence, it is plausible to observe the relative high internalization rate of rod-like micelles, as compared to spherical micelles. However the comb-like micelles were rarely employed to investigate the endocytosis, it’s hard to give a clearly explanation to the results. But we speculate that the size of the comb-like micelles maybe the main reason that embarrass their internalization.

**Figure 3:**
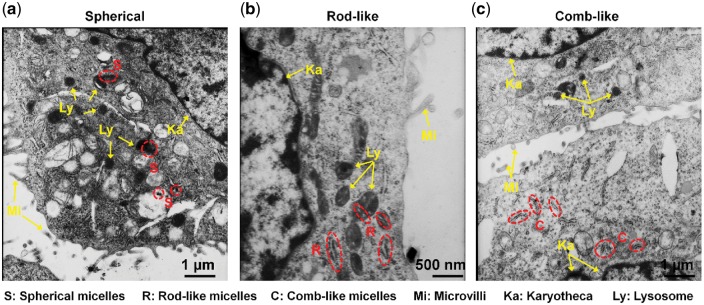
The high-resolution TEM images of ultrathin section of HeLa cells after 6 h incubation with (**a**) spherical (S), (**b**) rod-like (R) and (**c**) comb-like c micelles. The internalized micelles are shown in the red dotted line circles. Microvilli (mi), karyotheca (ka) and lysosome (ly) were pointed out by yellow arrows.

**Figure 4:**
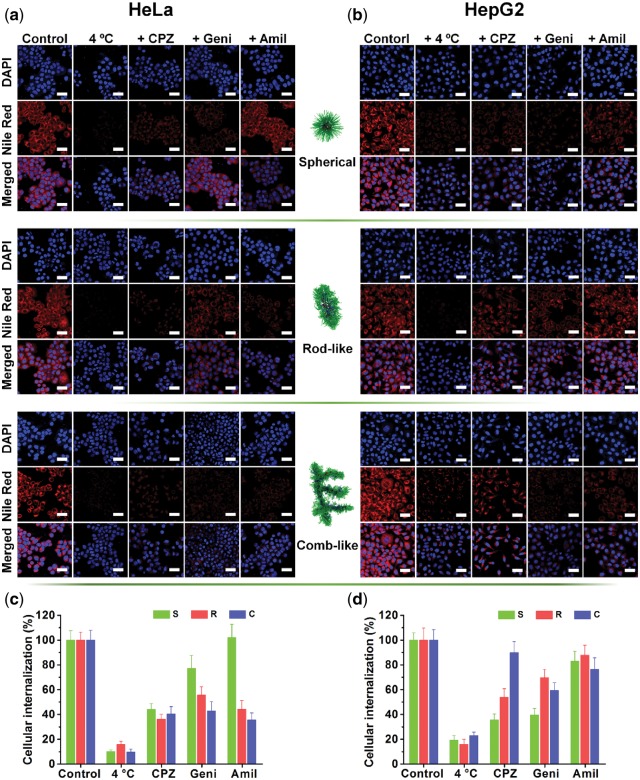
Evaluation of the endocytic pathways of spherical, rod-like and comb-like micelles in HeLa and HepG2 cells. **(a, b)** CLSM images of HeLa and HepG2 cells, respectively, incubated with spherical, rod-like and comb-like micelles for 3 h at different conditions, including: 1) 4 °C culture temperature only and 2) 1 h’s preincubation with different endocytic inhibitors (chlorpromazine (+ CPZ, 10 µg/mL), genistein (+ geni, 50 µg/ml), amiloride (+ amil, 13.3 µg/ml) and none (control)) at 37 °C, followed by another 3 h incubation. The nuclei were stained with DAPI (blue). the micelles were all labeled by nile red (red). the scale bars are 40 µm. **(c, d)** quantitative analysis of cellular internalization of different micelles in HeLa and HepG2 cells, respectively, in different conditions. Data is obtained by the image-pro plus 6.0 software.

Moreover, to gain a direct and detailed visualization of the cellular uptake and intracellular distribution of micelles, ultrathin section samples of HeLa cells, incubated with three types of micelles for 6 h, were viewed by TEM (Fig. 3). As marked in red dotted circles in the TEM images, all three types of micelles can be found in the intracellular space. Additionally, several spherical and rod-like micelles appear to be clumped (Fig. 4a and b) and trapped in one lysosome, respectively, suggesting that both of these two micelles are entering through clathrin-mediated endocytosis, since clathrin-mediated pathway is known to render the formation of primary endosomes, which consequently form late endosomes and lysosomes [25]. While, the comb-like micelles were not found to be separated by any lysosomes, which is consistent with the inhibition study.

### Insight into the cellular internalization of different architectures

To further understand why the relative internalization rates were different for each morphological type and to Fig. out the role of specific endocytic pathways involved in the internalization of the three types of self-assembled architectures, HeLa and HepG2 cells were further treated with potent biochemical inhibitors of chlorpromazine (CPZ, clathrin-mediated endocytosis inhibitor), genistein (Geni, caveolin-mediated endocytosis inhibitor), and amiloride (Amil, macropinocytosis inhibitor), and were also cultured at 4°C to show that the cellular internalization of micelles is an active and energy-dependent process [36].

As shown in Fig. 4a–d, culturing cells at low temperature (4°C) successfully inhibited the energy-dependent pathway, resulting in dramatic decrease in the cellular uptake rate of all three types of self-assembled architectures (75–90% decrease compared to control groups) against both cell lines. Next, the CPZ treatment of cells is known to perturb clathrin-mediated endocytosis, which caused a dramatic decrease (∼55, ∼62 and ∼60%) in the uptake of spherical, rod-like and comb-like micelles against HeLa cells, respectively. However, interestingly, the inhibition on the uptake of these three types of self-assembled architectures against HepG2 cells after CPZ treatment, shows a big difference, with a very uneven decrease (∼65, ∼45 and ∼7%) in the uptake of spherical, rod-like and comb-like micelles, respectively. Then, blocking caveolin-mediated endocytosis pathway by Geni, significantly decreased the uptake of rod-like and comb-like micelles for HeLa cells, exhibiting ∼45% (rod-like micelles) and ∼55% (comb-like micelles) decrease, but slightly decreased the uptake of spherical micelles for HeLa cells with only about 22% decrease. While for the HepG2 cells, the Geni shows a little weaker inhibition effect on the internalization of rod-like (∼30% decrease) and comb-like (∼38% decrease) micelles, but a stronger inhibition on the uptake of spherical micelles (∼60% decrease). At last, amiloride (Amil), an inhibitor of pinocytosis, did not show any obvious inhibition effect on the endocytosis for spherical micelles against the HeLa cells. However it exerts a significant effect on the uptake of rod-like and comb-like micelles for HeLa cells, with ∼55% and ∼65% decrease, respectively. For the HepG2 cells, Amil show a weak inhibition effect on the endocytosis for all types of micelles, namely, ∼15% decrease of spherical micelles, ∼10% decrease of rod-like micelles and ∼25% decrease of comb-like micelles.

In general, two important conclusions could be drawn from those results. First, none of the above specific chemical inhibitors led to over 70% inhibition of cellular uptake, which means these self-assembled architectures of PEG-CPP-SA may be uptake into cells through multiple endocytic pathways. Second, for the same type of self-assembled architectures of micelles, different cell lines may have different endocytic pathways. In detail, the spherical micelles mainly follow clathrin-mediated endocytosis for HeLa cells, but for HepG2 cells, clathrin- and caveolin-mediated endocytosis events are both prominent pathways for them. While, for the rod-like micelles, all the three endocytic pathways make a significant contribution to the uptake by HeLa cells, but they turned out to have an obviously weaker effect on the uptake when against HepG2 cells than that when against HeLa cells. Finally, in the terms of comb-like micelles, the three endocytic pathways also play an important role for the uptake by HeLa cells, but when against HepG2 cells, the clathrin- mediated endocytosis have only slightly effect and caveolin-mediated endocytosis and macropinocytosis have a weak effect. These results were also confirmed by flow cytometry (Supplementary Fig. S4).

### *In vivo* blood circulation experiments

**Figure 5:**
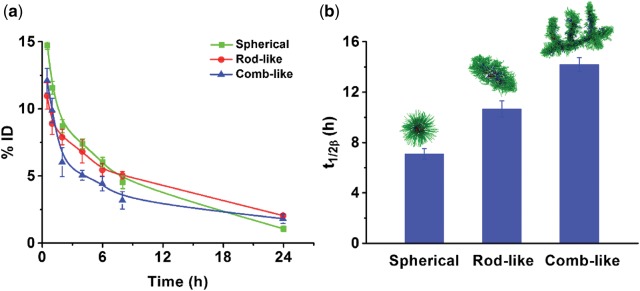
*In vivo* pharmacokinetics. (**A**) pharmacokinetic profiles of nile red after tail vein injection of various nile red labeled micelles. (**B**) the elimation half-lives of different micelles. (*n* = 3).

The *in vivo* blood circulation of the three types of micelles was studied after intravenous injection to healthy Balb/c male mice. From the blood clearance curves (Fig. 5a), we can find that the rod-like and comb-like micelles showed a relative lower content during the first 6 hours, but they were also eliminated relatively slower than spherical micelles. After 24 h, the content of spherical micelles was turned into the lowest one. The elimination half-life (t_1/2β_) (Fig. 5b) of comb-like micelles (∼ 14 h) was higher than that of rod-like micelles (∼ 10 h) and spherical micelles (∼ 7 h), which means the comb-like micelles possess more invisible performance than the other two micelles, exhibiting a relatively longer blood circulating feature. The possible reason maybe that the flexible, elongated and branched shape maybe help to increase the sensitivity to fluid forces, which allows them to tumble and align with the blood flow [37, 38].

## Conclusion

In summary, three types of polymer micelles with spherical, rod-like and comb-like architectures were successfully fabricated from the PEG-based ether-anhydride terpolymers (PEG-CPP-SA), by simply tuning the water addition rate during the self-assembly process. These three types of micelles provide prototype models for shape-regulated cellular internalization and blood circulation. The *in vitro* cellular uptake showed that the relative uptake rate was different for each morphological type, and multiple endocytic pathways contribute to their internalization, which was also affect by the cell types. The rod-like micelles, mediated into cells by clathrin-mediated endocytosis, caveolin-mediated endocytosis and macropinocytosis, possess the fastest internalization rate in both HeLa and HepG2 cells. The spherical micelles mainly follow clathrin-mediated endocytosis for HeLa cells, and followed clathrin- and caveolin-mediated endocytosis for HepG2 cells. The comb-like micelles showed the lowest internalization rate (might due to its larger size), but they displayed a longer blood circulating half-life than spherical and rod-like micelles.

## Supplementary Material

Supplementary Data
