# Supplementary material for: Polyanhydride micelles with diverse morphologies for shape-regulated cellular internalization and blood circulation
Source: Regen Biomater. 2017 Feb 4;4(3):149–57. doi: 10.1093/rb/rbw047 (PMC5458537; doi:10.1093/rb/rbw047)
Supplement: Supplementary Data [file rbw047_Supp.docx]

**Electronic Supplementary Information**

**Polyanhydride Micelles with Diverse Morphologies for Shape-Regulated Cellular Internalization and Blood Circulation**

Guang Yang, Jie Wang, Dan Li, Shaobing Zhou*

*Key Laboratory of Advanced Technologies of Material, Minister of Education, School of Materials Science and Engineering, Southwest Jiaotong University, Chengdu 610031, China*

*Correspondence address. Key Laboratory of Advanced Technologies of Material, Minister of Education, School of Materials Science and Engineering, Southwest Jiaotong University, Chengdu 610031, China. E-mail: shaobingzhou@swjtu.edu.cn, shaobingzhou@hotmail.com; Tel: (+86) 28 87634068; fax: (+86) 28 87634649.

## Supplementary Data


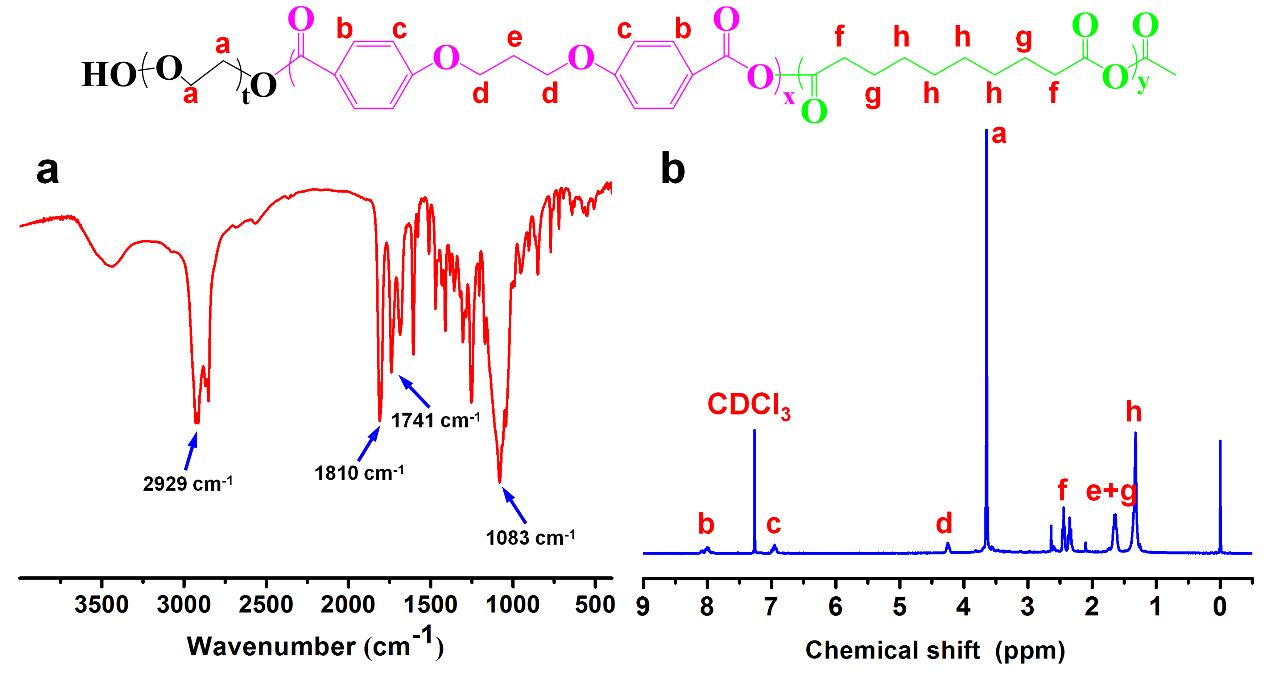


**Figure S1**. a) FTIR spectra and b) ^1^H NMR spectra in CDCl_3_ of PEG-CPP-SA copolymers.


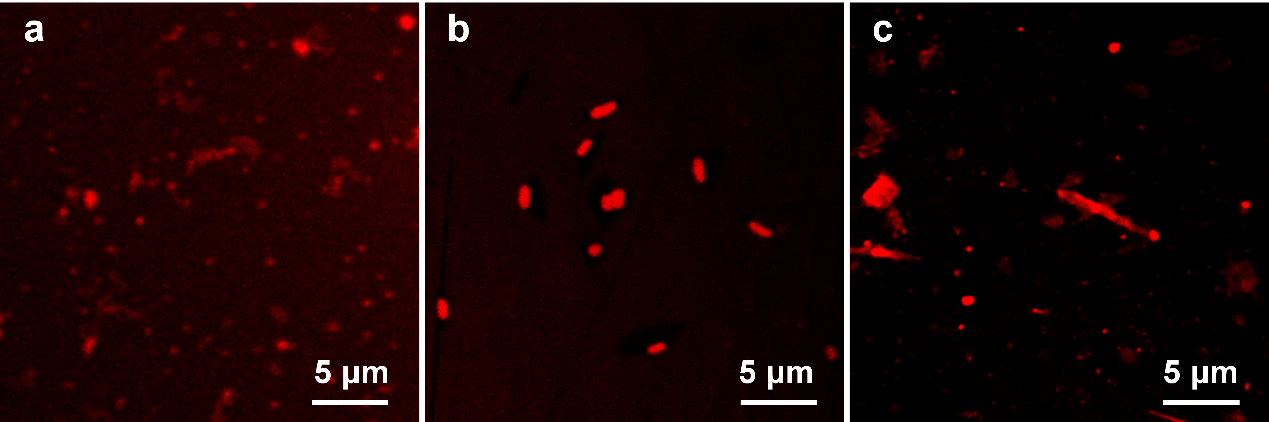


**Figure S2**. CLSM images of a) spherical, b) rod-like and c) comb-like micelles labeled by Nile Red.


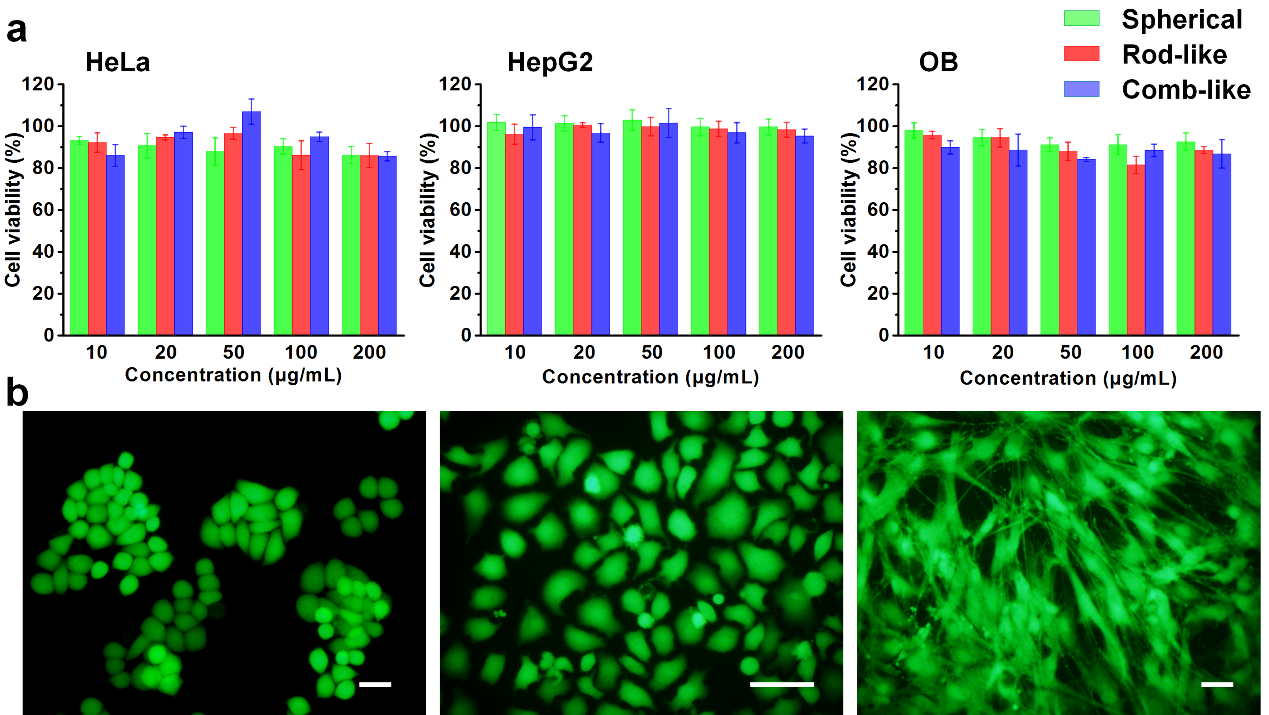


**Figure S3**. a) Cell viability of HeLa, HepG2 and OB cells incubated with spherical, rod-like, and comb-like micelles at different concentrations for 24 h. b) The corresponding Fluorescence images of HeLa cells, HepG2 cells and OB cells. Cells were stained by calcein AM (green).


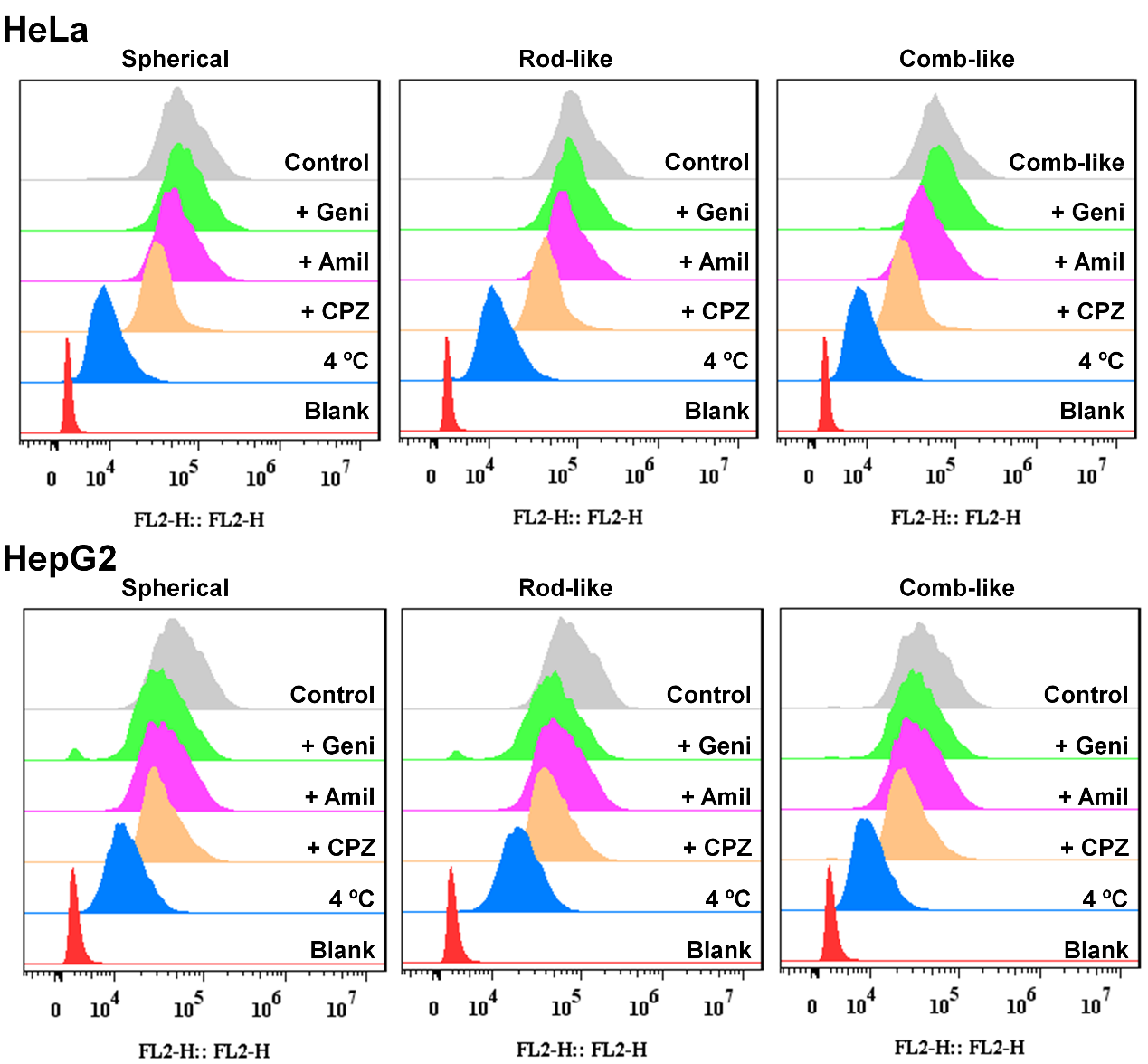


**Figure S4.** Effect of endocytosis inhibitors and culture temperature on the uptake of spherical, rod-like and comb-like micelles in HeLa and HepG2 cells measured by flow cytometry.
